# Problematic use of sustainability claims in recent scientific literature on crop gene technologies: toward improving practices and communication

**DOI:** 10.1111/tpj.70137

**Published:** 2025-04-12

**Authors:** Chris Wenzl, Emily A. Buddle, Rachel A. Ankeny

**Affiliations:** ^1^ School of Humanities The University of Adelaide Adelaide South Australia 5005 Australia; ^2^ Philosophy Group Wageningen University Wageningen The Netherlands

**Keywords:** sustainability, gene technologies, agricultural genomics, biotechnology, public understanding of science, science communication, crops, scientific impact, publication practices

## Abstract

There is growing interest in the role of agricultural genomics, including biotechnology, in enhancing the “sustainability” of food production systems. However, as “sustainability” becomes more frequently linked to the goals of agricultural genomics, a critical question arises: what claims are made about “sustainability” and how is the concept of “sustainability” defined in the scholarly literature on agricultural genomics? Using a structured analysis of the recent scientific literature, this article investigates increasingly frequent claims about “sustainability,” including how this term is defined and measured in the field of agricultural genomics. It argues that more transparent definitions and clearer metrics, tied to appropriate scholarly literature, are crucial for improving the coherence, impact, and credibility of research in agricultural genomics.

## Significance Statement

This article provides a structured analysis of the recent scientific literature to investigate increasingly frequent claims about “sustainability,” including how this term is defined and measured in agricultural genomics. It argues that more transparent definitions and clearer metrics, tied to appropriate scholarly literature, are crucial for improving the coherence, impact, and credibility of research in agricultural genomics.

## INTRODUCTION

There is growing academic and industrial interest in the role of agricultural genomics in enhancing the “sustainability” of food production systems. We use the term “agricultural genomics” (after Ruder & Kandlikar, 2023) to describe the field of biology that explores the structure, function, evolution, mapping, and editing of genomes for agri‐food applications. Agricultural genomics experts are usually trained in molecular biology or genetics, and their research includes experiments in lab settings, computational work, and sometimes fieldwork, using techniques including genetic modification and gene editing.

Sharma et al. (2022) argue that these types of biotechnologies hold promise for addressing a range of sustainability‐related domains, including climate change, food security, agriculture, forestry, food processing, and chemical manufacturing. However, as “sustainability” becomes more frequently linked to the goals of agricultural genomics, a critical question arises: what claims are made about sustainability and how is the concept of “sustainability” defined in the scholarly literature on agricultural genomics?

The concept of “sustainability” is widely used and deeply contested, making its meaning challenging to define. Ramsey (2015) highlights the inherent complexity of the term “sustainability” by describing it as a normative concept, meaning that it inherently reflects values, goals, and judgments about what ought to be sustained, how, and why. The term is not neutral; its meaning is influenced by the ethical framework or value system underlying its application, including the field or discipline in which the person making the claim is located. This inherent normativity leads to ambiguity in how “sustainability” is understood and applied, as different ethical and other perspectives tend to prioritize different values. As a result, claims about “sustainability” can be vague or misleading if the underlying ethical assumptions are not made explicit. Understandings of “sustainability” as a concept have evolved over time and across different disciplines. The adjective “sustainable” first appeared in the 17th century. However, the use of “sustainability” as a noun, vaguely defined as “capable of being continued at a certain level,” only came into use in the mid‐20th century (https://www.oed.com/dictionary/sustainability; Waseem & Kota, 2017). It has its roots in the German word “Nachhaltigkeit” which refers to forestry and is associated with harvesting only as much as a forest can regenerate (Kuhlman & Farrington, 2010). Over time, these terms migrated into environmental literature, where the term “sustainability” was used to describe systems, processes, and even societies that could be sustained indefinitely. Early usages highlighted the increasingly serious environmental challenges caused by human activities and the importance of balancing human interactions with their impacts on the world. As a result, theorists began advocating for adjustments to economic and population growth to ensure the protection of ecosystems (Attfield, 2023).

A major milestone in discussions of “sustainability” came in 1987 when the World Commission on Environment and Development (WCED) linked the concept to development, resulting in the widely used terminology of “sustainable development.” The publication of *Our Common Future*, also known as the Brundtland Report, defined “sustainable development” as meeting the needs of the present without compromising the abilities of future generations to meet their own needs (WCED, 1987). However, it is important to note that “sustainability” and “sustainable development” are not synonymous terms, and the latter has faced significant criticisms for its focus on development and/or economic growth at the expense of environmental and other considerations (Ruggerio, 2021).

“Sustainability” is now commonly viewed through three lenses: social, economic, and environmental. These perspectives are often referred to as the “three pillars of sustainability” or the “triple bottom line” (Purvis et al., 2019; Waseem & Kota, 2017) and represent distinct approaches to the concept, with varying definitions provided within each approach or point of view. However, this type of taxonomy has been criticized for neglecting political, cultural, and other dimensions of “sustainability,” as well as oversimplifying the relationships between the three subtypes by appearing to define them as mutually exclusive or siloed (Kuhlman & Farrington, 2010; Lozano, 2008; Purvis et al., 2019).

When applied to complex entities such as global food systems, the potential underlying meanings of the term “sustainability” become even more complicated: some forms of agriculture may be economically sustainable and contribute to social sustainability, especially in rural communities, but be in tension with common definitions of environmental sustainability, and vice versa. There is no widespread agreement on the best metrics for any one subtype of “sustainability” (Barry & Nelson, 2008), although measures such as carbon footprint and greenhouse gas emissions have become increasingly common in relation to environmental sustainability. Hence, it is critical when reading scholarly literature to ensure that claims made about “sustainability” are grounded in clear definitions and explications, particularly with regard to how “sustainability” is being assessed and measured.

When we refer to “claims,” we adopt a definition proposed by  who identify three primary types of claims in social science research, each with distinct characteristics and challenges. While these categories were originally developed for social science research, we argue that they also can be applied to our analysis of papers in agricultural genomics that make what we term “sustainability claims” as this taxonomy is usefully generic.

Using Gorard and Tan's (2022) framework, claims can be categorized as (1) descriptive, (2) general, or (3) causal:

1. Descriptive claims rely solely on observed data without making inferences beyond the dataset. For example, in the case of “sustainability,” descriptive claims might involve measuring emissions or other quantifiable data.
2. General claims extend beyond observed data, using inductive reasoning to generalize findings to broader populations or contexts. For instance, a general “sustainability claim” might be that specific interventions are associated with reduced emissions.
3. Causal claims assert that one variable or event directly influences or causes another. These claims combine descriptive and general elements while attempting to establish causation. For instance, in the context of “sustainability,” a causal claim might state that certain interventions directly lead to reduced emissions.

Without providing evidence about a cause‐and‐effect relationship, terms such as “sustainability” risk being reduced to mere “buzzwords” (Søilen, 2020) which only support weaker general claims (see also Shao, 2024). Furthermore, proper citation practices are fundamental for upholding the integrity and rigor of academic work and are essential in domains where terms may have multiple meanings or require further definition, such as when using the term “sustainability.” Hicks (2021) underscores the critical role of accurate citations in ensuring academic integrity, credibility, and evidence‐based practice, additionally cautioning against reliance on secondary sources. Lin et al. (2022) explore missing citations for well‐known concepts in computer science, highlighting a tendency to omit proper attribution concerning what we call “buzzwords” in this paper and urging better citation practices to maintain academic rigor including explicitly crediting foundational works.

This article provides a structured analysis of the recent scientific literature in the field of agricultural genomics to investigate claims about “sustainability,” including how this term is defined and framed in the context of crop production. This topic is important to explore as it connects closely to the promises being made in agricultural genomics. Scholarly literature is often used as the basis for media coverage and public engagement in science, and hence it is critical to assess how this literature views the contributions of this type of research to “sustainability.” In addition, given the increasingly interdisciplinary nature of many fields including agricultural genomics, fostering clear communication within and across various scientific fields is essential.

## METHODS

A Systematic Literature Review (SLR) method (Hempel, 2020) was selected because it provides a structured and comprehensive approach to systematically collecting recent scientific literature in this field and extracting data from it. We then used critical content analysis to explore claims made about “sustainability” within the context of journal articles. Content analysis is a research methodology that allows the determination of the presence of certain words, themes, or concepts within some set of texts (in this case, published journal articles). The aim of the study was not to provide frequencies of usage of this term (or related ones such as “sustainable”). Instead, we aimed to assess how the articles defined and referenced “sustainability” in the context of claims made about it, offering insights that would not have been revealed via a purely quantitative approach. Our focus in this paper was on scientists working in part or whole within public institutions (and acknowledge that there might be distinct and sometimes competing responsibilities associated with claims made by those doing research solely within industry; cf. GTECCC, 2024). Hence, published scholarship provided an appropriate set of texts for this analysis.

### Search strategy

To conduct the analysis of sustainability claims in agricultural genomics, we applied a well‐defined, reproducible search strategy (see Appendix S1) to two major databases, Web of Science (WoS) and Scopus. These two databases were used to maximize the size and relevance of the dataset of journal articles since they cover slightly different sets of journals and research articles. We employed Boolean logic to construct the search strings, using combinations of relevant terms for gene technologies, sustainability, and agriculture‐ and crop‐related keywords. The search terms were adjusted to fit the specific structure and syntax of each database, ensuring comprehensive coverage of the relevant literature. The search was restricted to English‐language scientific journal articles published between January 1, 2012, and December 1, 2024, which allowed us to trace changes in usage and capture recent scholarship associated with developments in agricultural genomics, particularly in light of new technologies such as CRISPR/Cas9‐mediated gene editing.

### Inclusion and exclusion criteria

In total, 5816 articles were identified from the searches performed across both databases. After eliminating duplicates using DOI comparison, 4159 unique articles remained. These articles were manually reviewed to ensure they met our inclusion criteria: scholarly articles focused on agricultural biotechnologies used in plants or crops associated with food production or consumption and explicit discussions of “sustainability” within the article. Claims related to the noun “sustainability” and the adjective “sustainable” (when paired with other relevant nouns such as “agriculture”) were identified via the search string “sustainab*”; henceforth for brevity, we refer to these as “sustainability claims.” One limitation of this methodology is that it relies on the explicit use of the term “sustainability” or “sustainable” as an adjective. We were not able to capture papers that did not make such explicit claims but may have presented data related to different forms of “sustainability” without explicitly using this terminology. However, this limitation was considered to be acceptable given that our research question was focused on explicit uses of sustainability claims.

**Figure 1:**
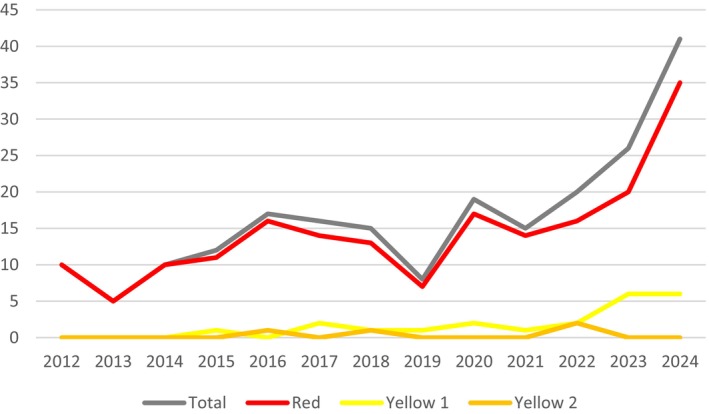
Flowchart of article selection process. *Source for flowchart*: Page et al. (2021).

The corpus was further filtered to ensure relevance to our main research question, resulting in a set of 214 articles that underwent manual, full‐text review for key characteristics and analysis. The selection process is depicted in a flowchart (Figure 1) based on the PRISMA guidelines for systematic reviews (Page et al., 2021). The review for inclusion in the dataset was performed by one author (C.W.) with a later validity check (Pyett, 2003) by a second author (E.A.B.).

This review focused on scholarly research in the agricultural genomics space, specifically examining how articles made sustainability claims about crops for food production and consumption. Articles were assessed based on the following inclusion and exclusion criteria:

1 Type of publication

To ensure the review included original, peer‐reviewed research, the following types of publications were excluded:
  - Summaries or reviews
  - Perspectives and opinion pieces
  - Book reviews
  - Other non‐research publications
Reviews, for example, were excluded because they did not provide firsthand data or text from scientists directly using gene technologies on crops.

2 Scope of research

Articles were excluded if they did not focus on natural scientific research directly related to gene technologies for crops. Thus:
  - Research focused on regulatory, political, economic, or social issues was excluded, as these were outside the scope of this review.
  - Studies using in silico methods or machine learning were also excluded, as they represent a distinct area of study that is outside the scope of this review.

3 Focus on human food plants

The review only included articles addressing human food crops. Excluded articles addressed:
  - Gene technologies in non‐food organisms, such as animals (including insects considered as “crop pests”), animal feed, soil, or bacteria.
  - Non‐food plant applications, including crops for biofuel production, plant model organisms, cannabis, tobacco, eucalyptus, and rubber, even if digestible.
  - Extractions from plants used as cosmetics, fragrances, vitamins, medicines, or vaccines.
  - Mulberries, as their modification was intended for increased silk production in silkworms, not human food consumption.

4 Relevance to gene technologies

Papers were included only if they discussed the use of gene technologies, such as gene modification or editing. Excluded papers focused on:
  - Pure genetics research, such as studies of wild plants, transcriptomics, metabolomics, or epigenetics, without any gene modification or editing.
  - Comparative studies between unmodified crops.
  - The use of fermentation for food production.

5 Specific inclusions

The review included articles on the use of gene technologies for specific food‐related products, including:
  - Grapes (primarily for wine production, but grapes and raisins still enter food chains).
  - Coffee and tea.
  - Oils intended for human consumption, including cottonseed oil from cotton.

### Data extraction and synthesis

For each journal article selected for inclusion, key information was extracted from the relevant database, including full citation details, the geographic locale of the first author, and text associated with any sustainability claims (including the broader context of the text surrounding the claims). Data were recorded in an Excel spreadsheet to allow comparison, analysis, and verification.

### Analysis via TLRS

We considered using traditional thematic analysis to explore sustainability claims in the literature sourced; however, it quickly became apparent that there was limited material available for analysis related to most of the sustainability claims (see “Results” section).

Thus a TLRS was developed and employed as an alternative method for summarizing the data, with the initial coding applied by one author (C.W.) with a later validity check (Pyett, 2003) by a second author (E.A.B.). In general, TLRSs use a color‐coded classification technique (red, yellow, and green) to evaluate the performance of the unit (in this case the journal article) analyzed against specific criteria (the robustness of sustainability claims made in each article). Such systems are commonly employed in project and management fields to simplify complex or larger datasets, enhance visualization of results, and foster clear communication about results. For instance in food labelling, a TLRS is used to indicate high (red), medium (yellow), or low (green) levels of fat, sugar, and salt (Food Standards Agency, n.d.). TLRSs have recognized limitations, including limited flexibility once categories are defined (Anonymous, n.d.; Schleutker, 2023). However, this methodology was deemed to provide a good starting point for the current analysis. After carefully analyzing the data through a structured analysis of the relevant literature, we modified the TLRS to have four categories which are defined in Table 1. A generalized example derived from the data is provided in the table to illustrate each category.

**Table 1:** Four categories used for analysis

Green	The article cites a credible academic source that defines the concept of “sustainability” when it is used. The cited academic source provides details about how this definition of “sustainability” should be interpreted or translated when applied in other contexts. The article explicitly discusses how the chosen definition of “sustainability” aligns with the research aims and findings of the current paper, highlighting tangible or measurable qualities or quantities that can be verified using scientific methods Example: Improving [a specified aspect of] crops using [specific types of] biotechnological tools is crucial for enhancing [tangible quality or quantity of] sustainability. [Conventional crops/tools] have [certain specified] negative side effects or limitations, which our modified crop aims to address, ultimately supporting the sustainability of [the crop in question]. (accompanying citation of an academic source that defines “sustainability” as a concept)
Yellow 1	The article does not cite a credible academic source that defines “sustainability” as a concept. Instead, it offers a general interpretation of “sustainability” within the context of the paper, presenting tangible or measurable qualities or quantities that can be verified or falsified Example: Improving [a specified aspect of] crops using [specific types of] biotechnological tools is crucial for enhancing [tangible quality or quantity of] sustainability. [Conventional crops/tools] have [certain specified] negative side effects or limitations, which our modified crop aims to address, ultimately supporting the sustainability of [the crop in question]. (no citation provided)
Yellow 2	The article cites a credible academic source that defines “sustainability” as a concept. The cited academic source provides details about how this definition of “sustainability” should be interpreted or translated when applied in other contexts. However, the article does not address how the selected definition of “sustainability” aligns with the research aims and findings of the current paper, nor does it provide tangible or measurable qualities or quantities that can be verified using scientific methods Example: Improving [an aspect of] crops using [certain] biotechnological tools is crucial for enhancing [a tangible quality or quantity of] sustainability. (academic source that defines sustainability as a concept is cited)
Red	The article neither cites a credible academic source defining “sustainability” as a concept nor provides a general interpretation of “sustainability” situated within the context of the paper. As a result, it does not present tangible or measurable qualities or quantities that can be verified using scientific methods Example: Improving [an aspect of] crops using [certain types of] biotechnological tools is crucial for enhancing sustainability. (no citation provided)

Note that these examples are generic and not actual citations from our dataset, as the intent of this paper is not to criticize specific articles but to raise awareness about the issues revealed in this analysis.

Interestingly enough, there are limited definitions in the scholarly literature about what counts as a “credible academic source.” Hence we use a common‐sense definition and view credible sources (for purposes of citation in scholarly scientific literature) as peer‐reviewed scholarly papers or major policy documents that provide a detailed understanding of the concept in question (in our case, “sustainability”), including research or review articles, which are readily accessible online (given that a key aspect of credibility is that others can access and read the source).

This taxonomy is not intended to be used as a checklist but rather was used as a guide for the analysis of the papers in this systematic literature review. The example sentences provided above are intended to be adapted to fit the specific context of the scientific study being conducted and should be seen as a guide rather than a strict template; we encourage deviation from these examples as required in any specific context although citations to relevant sources and connecting that definition to the work described are obviously critical components of any sustainability claim. Our aim is not to critique the quality of the science in the studies analyzed but to emphasize the need for greater awareness about how sustainability arguments are presented in scientific discourse. Without explicit definitions and clear metrics – both qualitative and quantitative – how can scientists assert that the modified crops discussed in the papers are going to improve “sustainability,” and how will these claims be convincing to others?

## RESULTS

**Figure 2:**
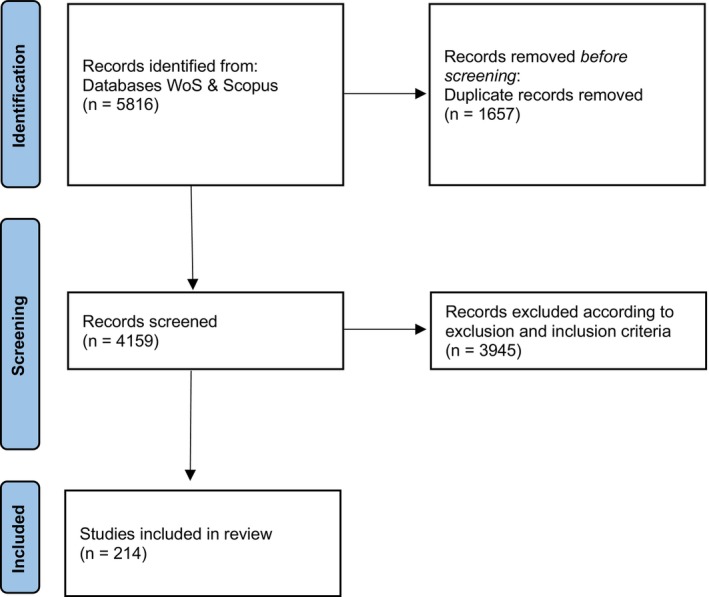
Bar chart illustrating the classification of articles in relation to their definitions of “sustainability” using a Traffic Light Rating System (TLRS).

Of the 214 articles reviewed, none received a green rating using the Traffic Light Rating System (TLRS) developed for this analysis as described in our Methods below (Figure 2). A total of 26 articles were in the yellow category, with 22 in the Yellow 1 category and 4 in Yellow 2. Most articles were in the Red category (188 articles).

Hence, the overwhelming majority of articles did not cite a credible academic source defining “sustainability” as a concept nor provide a general interpretation of “sustainability” within the context of the paper. As a result, these articles do not present tangible or measurable qualities or quantities that can be verified, despite making claims about “sustainability.” Based on Gorard and Tan's (2022) definition of claims discussed above, our analysis found that most sustainability claims in the corpus fall into the causal claim category. These types of claims typically aim to establish a cause‐and‐effect relationship, such as asserting that the use of gene technologies in crops will achieve specific or unspecified sustainability goals.

It is important to note that the use of the term “sustainability” as a standalone term and claims associated with it tended to imply a general concept, while the use of the term “sustainable” occurred in conjunction with specific relevant nouns such as “agriculture” or “strategy” and tended to highlight targeted applications or goals within particular contexts. However, the lack of precise definitions and associated credible references was notable in both types of uses of terminology related to “sustainability.”

**Figure 3:**
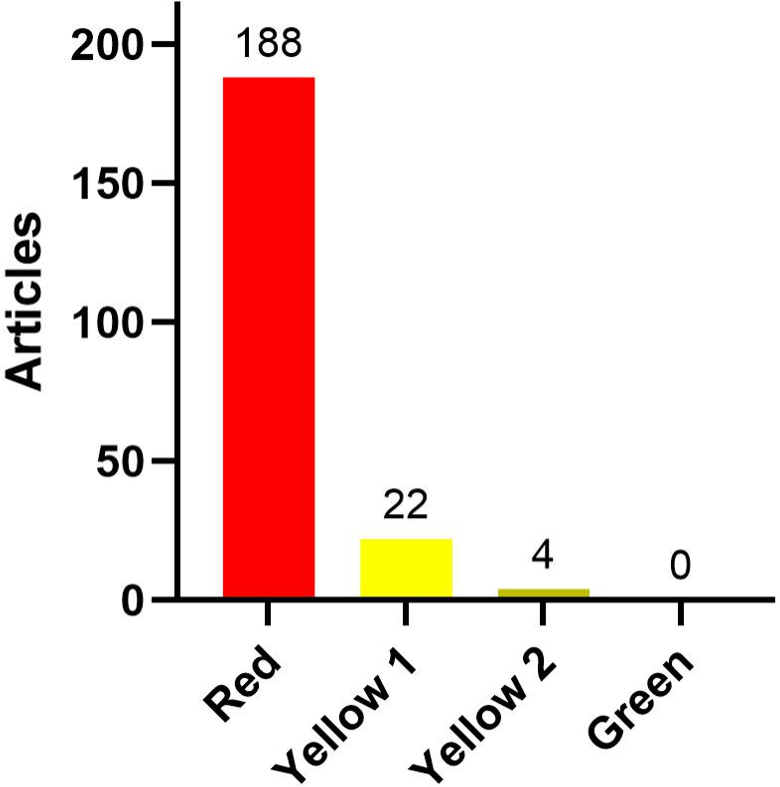
Line chart depicting the trend of article growth over time, along with the distribution of red, yellow 1, and yellow 2 categorization, analyzed using a TLRS.

We developed several hypotheses for this absence that we tested using the dataset generated and our analysis of it. First, during the early years of the period in which the scientific literature was surveyed, there may have been less attention to defining “sustainability,” but practices could have evolved in recent years due to increasing attention to the concept and its complexities in different contexts. However, despite a general trend of an increasing number of publications making sustainability claims in the dataset between 2012 and 2024 (with notable dips in numbers in 2013 and 2019 that are difficult to explain), there was no notable increase in the inclusion of definitions of “sustainability” (i.e., more yellow than red ratings) over this period (Figure 3).

Second, it might be hypothesized that the quality of the journal might affect whether explicit definitions of “sustainability” were provided. However, the journals in this dataset were predominantly Q1, a rating that is supposed to reflect a relatively high level of quality and rigor, including in the peer‐review processes, as well as likely higher impact in their field. To assess journal quality, we utilized the SCImago Journal and Country Rank, a publicly accessible portal that provides scientific indicators for journals and countries based on data from the Scopus^®^ database (SCImago, n.d.). Furthermore, there were no discernible differences in TLSRs in relation to the quartile of the journal in which an article was published.

A third hypothesis was that different disciplinary traditions might have diverse practices of defining terms and providing citations: for instance, perhaps those working within the same discipline might assume that they share the same or similar definitions of “sustainability” with others in their field (and hence not explicitly define the term in their publications), while those in more interdisciplinary fields or publishing in journals coded as interdisciplinary or multidisciplinary might tend to provide explicit definitions in conjunction with sustainability claims because the journal's readership would be assumed to be more diverse. This hypothesis was explored using coding by discipline of the journals that contained the articles utilized to create the dataset: all articles reviewed fell under the natural sciences, with 33.64% (72 articles) from Plant Sciences and 9.81% (21 articles) from Biotechnology and Applied Microbiology with other disciplines having smaller percentages; 13.08% (28 articles) were classified as Multidisciplinary Sciences. Our review showed that “sustainability” tended to be equally infrequently defined across all disciplines (i.e., some yellow but mostly red ratings, with no green ratings). Interestingly, although we predicted that those classified as belonging to Multidisciplinary Sciences might have slightly better results than single‐discipline papers, given that they were more likely to involve a multidisciplinary team that might have been predicted to have engaged in more explicit discussions about terminology and key concepts, 78.57% of articles in this classification received a red rating, indicating no definition or citation whatsoever in relation to their sustainability claims.

Finally, another factor hypothesized to have a potential relationship to patterns of definitions (or lack thereof) was the location of the first author, as certain locales might have tendencies to make assumptions about shared understandings about how “sustainability” should be defined or conceptualized. The articles included in this analysis originated from various research institutions worldwide, led by China (74 articles); India and the United States (between 25 and 30 each); Australia, Brazil, Pakistan, and Switzerland (8–10 each); and all remaining countries (5 or less each). However, the location of the first author did not correlate significantly with particular definitional practices based on the analysis: as noted above, no article was classified as green, and the distribution of yellow and red ratings was proportionate to the number of articles without significant differences amongst the countries of the first author. A more robust analysis of this factor would require determining the location of all authors and considering how authorship networks or even types of institutions may have affected authors' practices associated with sustainability claims. However, given the relatively limited evidence of explicit definitions of “sustainability” across the entire dataset, such an analysis was deemed to be unlikely to result in any useful findings and thus was not pursued.

It is important to note that when we developed our research questions, we had hoped to explore not only how “sustainability” was being defined in relation to sustainability claims, but also how each research paper proposed to measure the potential impact of its findings in terms of the sustainability claims made. So, for instance, if a paper provided evidence of a new genetically edited strain of a particular crop designed to increase salinity tolerance, claims could be made with regard to the potential effects of such a strain on rates of crop growth and productivity, and hence, its contributions to economic sustainability could perhaps be quantified in relation to the baseline of current, less saline‐resistant strains. Alternatively, this type of strain might be argued to be more environmentally sustainable as compared to strains currently in use in light of documented changes to soil and other environmental factors, with quantitative and/or qualitative data provided to substantiate this type of claim.

This consideration led us to perform a further analysis of where sustainability claims were made in the context of the structure of each published paper: it was striking that the claims most frequently occurred in the papers' abstracts (222 articles), introductions (114), and conclusions (99), noting that the total exceeds the total number of articles as some articles contained multiple claims. It was especially notable that sustainability claims only occurred in the body of the paper (including methods, results, and/or discussion sections) in 16 of the papers in the dataset. We would have expected discussions of qualitative or quantitative metrics or measures related to “sustainability” to occur in the body of the paper, and thus, these results reinforce our finding of limited explicit engagement with explicit definitions of “sustainability.”

## DISCUSSION

The analyses presented in this paper are not intended as simplistic or deflationary criticisms of the articles analyzed, their authors, the journals in which they were published, or the labs or groups from which they originated. Instead, the goal of this paper is to draw attention to the complexities associated with making claims about rich and contested concepts such as “sustainability,” and to provide a series of considerations for scientists working in agricultural genomics about how they can improve their practices when writing (and talking) about the potential impacts and applications of their research in this rapidly moving field and in science more broadly.

Hence, we provide four lessons for researchers in agricultural genomics based on our analysis: (1) think about your rhetoric, especially in publications and communications that are likely to travel beyond the boundaries of science into public arenas; (2) reflect on approaches to improving accessibility to these multiple audiences; (3) establish shared best practices for making claims about “sustainability” particularly in scientific publications; and (4) make scientific claims more transparent and robust in research publications in particular through more collaboration. We believe that greater consideration of these issues is critical for fostering more robust public and broader scientific engagement with developments in agricultural genomics, particularly in light of the widespread desire to build greater trust in the science associated with this field.

### Lesson 1: think about your rhetoric

When communicating about science and technology via scientific publications or otherwise, it is important that language and rhetoric are clear and claims are accurate regardless of the specific audience to which the communication is directed (Medvecky & Leach, 2019). A skeptic might suggest that anyone reading articles in a particular journal or field will “know” what type of “sustainability” is being discussed or being argued to be targeted by the research in question. However, given the multiple disciplines that contribute to research in agricultural genomics (e.g., molecular biology, agriculture, plant physiology, and crop science, to name just a few), these types of assumptions are not well grounded. Making claims with reference to complex and contested concepts such as “sustainability” without providing explicit definitions or metrics creates barriers to fostering understanding within and across disciplines and introduces opportunities for misinterpretation.

As more generally is the case with the use of scientific jargon, scientists (perhaps unconsciously) exclude non‐experts in their specific field by making claims that rely on unarticulated assumptions. In turn, such practices may result in a loss of trust in this type of research as non‐experts may think that such claims are unclear or highly speculative, and hence dubious. Given the increasing pressure for researchers to make their publications open access, the potential audience for scientific publications has widened well beyond their discipline of origin. In the domain of agricultural genomics, this newly broadened audience may well include policymakers, educators, industry professionals, non‐governmental organizations, farmers and others involved in agriculture, and broader publics who are likely to lack the specialized knowledge to understand all of the details associated with the research, but who will recognize sustainability claims and potentially weigh them up against the evidence presented.

Enabling these groups to better understand scientific research and developments is a powerful way to help them to make better decisions, develop reasoned attitudes for or against technological developments, and ultimately live better lives (Rice & Giles, 2017). Careful communication also has been argued to facilitate the resolution of public controversies, enable mutual learning, and build capacities within publics to deliberate on evidence and make well‐grounded policy decisions (Scheufele et al., 2021; for a review of empirical evidence about science communication, see Kappel & Holmen, 2019). Communicating clearly and with integrity is particularly important with reference to gene technologies given the broad and wide‐ranging effects that such technologies could have (Gene Technology Ethics and Community Consultative Committee [GTECCC], Office of the Gene Technology Regulator, Commonwealth of Australia, 2024), and the concerns that some publics have about their use, especially in agriculture and food production (see also Baik et al., 2022). Some have argued explicitly that ethically responsible uses of agricultural biotechnologies require effective science communication (Harfouche et al., 2021). The ability to effectively communicate about research, including underlying assumptions, potential limitations, and uncertainties, will enhance the reach and impact of research as well as have the potential to improve interdisciplinary engagement amongst scientists and public trust in science.

### Lesson 2: reflect on improving accessibility

Accessibility is in itself critical to consider in this context. Tattersall (2015) explores the “5 Ws” framework (Who, What, Where, When, Why) as a tool for making academic research more accessible to diverse audiences. They claim that this approach not only simplifies research communication but also enhances its broader impact, allowing researchers to maintain control over their narratives. This method is particularly valuable for academics aiming to engage effectively with non‐specialist audiences. Dabbicco et al. (2023) extend this framework to include the “How,” creating the 5W+H model. They present it as a narrative structure for effective science communication and education, emphasizing the need to adapt the sequence of these elements to suit different audience needs. The authors underscore the importance of the “How” as a critical concluding element for fostering clarity and accessibility.

We contend that both frameworks have further applications beyond their original contexts. In the current context, they can be leveraged by scientists to enhance the clarity and tangibility of sustainability claims in research papers and related communications, providing a structured yet flexible approach to presenting complex ideas to both academic and non‐academic audiences.

The questions can be tailored by researchers to effectively highlight and embed sustainability claims across disciplines in an accessible manner, but they can be broadly framed as follows:

- WHAT is the context‐specific interpretation of “sustainability” in this research?
- WHY can this research be considered to promote “sustainability”?
- WHO stands to be impacted by this research?
- WHERE is this research expected to have an impact?
- WHEN are these impacts anticipated to be realized?
- HOW is this research aligned with the selected definition or concept of “sustainability”?

Each of these questions can be further explored via follow‐up questions such as: are there any trade‐offs to consider? By addressing these questions, researchers can gain a comprehensive understanding of the scope and significance of, for example, crop modification within a framework associated with “sustainability.”

### Lesson 3: establish shared best practices

Scientists working in agricultural genomics should consider ways to establish shared standards of best practice for scientific publications and other forms of communication in relation to claims about complex and contested concepts such as “sustainability.” The intention of such practices is not to legislate how words should be used or to provide one agreed definition for commonly used terms but instead to establish guidelines for scientists to consider when making claims about such concepts and for reviewers to use when assessing arguments and evidence in publications, grants, and other forms of communication.

A notable finding in our analysis was that sustainability claims rarely occurred in the body of papers included in the dataset, and instead tended to be present in the introduction, conclusion, and/or abstract. This pattern suggests that sustainability claims have not been adequately integrated into the methods, objectives, findings, or arguments in these papers but are being used to grab readers' or reviewers' attention, or perhaps are carry‐overs from broader descriptions of project or laboratory goals in other contexts such as grant applications.

Establishing and following best practices ensures research and the claims in it are credible and transparent, allowing confidence and trust to be built amongst other scientist‐readers, policymakers, and publics, and reducing risks associated with overpromising or relying on buzzwords. Researchers should ensure that they are not making ill‐founded claims to justify their research, particularly when it is in relatively early stages. For example, to claim that using CRISPR to transform Arabidopsis to be more drought tolerant in a laboratory setting is important for agricultural sustainability in a certain locale without any further information about what is meant by “sustainability” or without evidence to support the connections implied by the claim (e.g., that this transformation can made in an actual crop plant with particular effects in a real‐world agricultural setting, and that drought tolerance is important for agricultural sustainability as defined in the paper in the locale in question) could be argued to be overstating the research results. In short, typical scholarly and scientific standards for evidence should be used whenever claims are made, particularly in relation to complex concepts such as “sustainability.”

With regard to grant applications, both researchers and grant funders contribute to how arguments are expressed and defended. In many schemes, scientists are required to describe the potential impacts that the proposed work may have within and beyond their home scientific discipline in order to defend the research's importance and benefits. Broad terminology that intersects with societal priorities such as “sustainability” often is used in this context and can result (once again) in overpromising. Grant funders, governmental entities, and research and developmental organizations should not encourage speculative claims when developing guidelines for such schemes and instead should stress the need for use of well‐defined, defensible, and evidence‐based terminology and arguments, particularly with regard to claims about research impact. Using more transparent definitions and metrics for claims associated with “sustainability” would help to strengthen the impacts of research in agricultural genomics, grounded in shared social, environmental, and scientific priorities.

### Lesson 4: make scientific claims more transparent via interdisciplinary collaboration

Transparency is a notoriously complex concept (Fischhoff, 2019). Scientists are often taught that data should be presented without interpretation and that the meaning of data is self‐evident (Smith & Merkle, 2021), and hence they have deeply grounded habits associated with how they write, communicate, and argue, particularly about their research findings. However, there is evidence that this type of approach to presentation of data is unrealistic: as in any other communicative endeavor, meaning is constantly shaped and reshaped by choices made about language and written expression. Political, social, cultural, economic, and ethical concerns impact and are impacted by science communication (Gigante, 2018), which begins with word and concept choice (Smith & Merkle, 2021).

Providing definitions and metrics for claims used in publications will allow scientists to recognize the limits and prospects of their research at various shorter‐ and longer‐term timepoints, and to allow others to get a window into these often extended processes. Making grandiose claims about the potential application of research and its likely contributions to broader societal goals such as “sustainability” leaves researchers in agricultural genomics open to criticism, especially when presenting delimited and/or only lab‐based data. Being transparent and specific about claims including the evidence and metrics utilized can allow others to more productively engage with the research, particularly if results are unexpected.

It is important to note that we are not proposing that scientists should abandon all attempts to link their research to relevant societal goals beyond the pursuit of scientific knowledge. There are numerous reasons why it is important to keep such broader goals in view, not the least of which is that much research in agricultural genomics is publicly funded. We *are* claiming that this type of research presents important opportunities for those working in agricultural genomics to more actively engage with scientists from different disciplines and others with appropriate expertise to better assess and develop claims, metrics, and evidence related to the potential application of their research (e.g., environmental scientists about environmental sustainability, social scientists and farmers themselves about rural community sustainability, and so on).

Such approaches are aligned with what has been argued to be one of the guiding ethical principles associated with science communication, generosity, which holds that other parties have their own forms of worthwhile knowledge, experiences, and aspirations that can make useful contributions to scientific research (Medvecky & Leach, 2019). Collaborating with scholars from other disciplines can assist those working in agricultural genomics to consider the broader social, cultural, economic, and environmental contexts within which their research is occurring in order to help shape their claims, particularly as these contexts are likely to be rapidly evolving.

## CONCLUSIONS

This article provides an analysis of sustainability claims made in recent scholarly publications in agricultural genomics. In short, the term “sustainability” is often used as a buzzword rather than a scientific concept (hence, the articles in which these claims appeared were classified as red in our analysis), or used uncritically without reflecting on implications or metrics associated with the definitions utilized (with articles of this type classified as yellow). Our analysis shows that although claims about “sustainability” are relatively common, more explicit definitions and clearer metrics for how “sustainability” is being fostered in the research discussed are crucial for improving the coherence, impact, and credibility of research in agricultural genomics.

Although we have focused on sustainability claims, the arguments made here have relevance to other claims frequently made in this domain using complex and contested concepts, including efficiency, climate resilience, food security, and many of the sustainable development goals (United Nations, n.d.), all of which have diverse definitions and metrics depending on context. Our findings (and methods of analysis) also are likely to be applicable to disciplines and fields beyond agricultural genomics, especially given increasing pressures to tie even blue‐skies or fundamental research to societal goals and impacts.

It is important to note that this paper is not intended as a critique of the quality of the science in these studies but instead is a call for greater awareness of how sustainability arguments are framed and presented in scientific discourse, reflection on how these practices can be improved, and changes to publication and funding norms with regard to the use of sustainability claims. Additionally, the results of this paper do not provide a basis for comparisons between disciplines about their use of sustainability claims, as this analysis is not intended to be an exercise to identify which discipline is performing in ways that are “right” or “wrong” but rather is designed to serve as an informative case of how academic publishing can be improved. Increasing focus on “sustainability” in a variety of senses underscores the need for explicit definitions grounded in credible references and appropriate metrics whenever presenting research in agricultural genomics and beyond. Addressing this current gap in scientific practices will result in significantly enhanced communication of research findings; more realistic goal‐setting about impacts and benefits both for claims within grants and similar but also within the research design itself; and more productive collaborations between diverse types of scientists as well as policymakers and publics, particularly as shared concerns associated with climate change (and relatedly “sustainability”) continue to grow.

## CONFLICT OF INTEREST

All authors are affiliated with the ARC Training Centre for Future Crops Development (IC210100047) (https://futurecropscentre.edu.au) as part of Program 2: “Develop responsible research innovation practices” and receive research funding from it. C.W. and E.A.B. receive partial salary support from the Training Centre. R.A.A. is a member of the Gene Technology Ethics and Community Consultative Committee (GTECCC), which is an advisory committee associated with the Australian Commonwealth Gene Technology Regulator (OGTR) and of the Social Science and Economics Advisory Group (SSEAG) for Food Standards Australia New Zealand (FSANZ). She was a lead contributor to the 2024 GTECCC Guidance Paper cited below. She is a recent past member of the GM Crop Advisory Committee, Government of South Australia, and E.A.B. is a current member.

## Supporting information


**Appendix S1.** Search strings.

## Data Availability

The data associated with this article can be accessed by contacting the corresponding author but will be embargoed until completion of the PhD thesis with which these data are associated.
