# Supplementary material for: Problematic use of sustainability claims in recent scientific literature on crop gene technologies: toward improving practices and communication
Source: Plant J. 2025 Apr 12;122(1):e70137. doi: 10.1111/tpj.70137 (PMC11992966; doi:10.1111/tpj.70137)
Supplement: Supplementary file 1 — Appendix S1. Search strings. [file TPJ-122-0-s001.docx]

**Appendix S1: Search Strings**

*Web of Science:*TS=((sustainab*) AND (“gen* engi*” OR “gen* manipulat*” OR “gene tech*” OR “gen* modif*” OR “gen* edit*” OR "gen* engineer*" OR "genetically modified organism*" OR "genetically engineered organism*" OR transgenic OR cisgenic OR crispr* OR GMO OR rna*) AND (enhanc* OR improv* OR effic* OR innov* OR fortif* OR secur* OR mitigat* OR adapt* OR develop* OR resist* OR control* OR introduc* OR alter* OR creat* OR appl* OR tolera*) AND (agri* OR farm* OR crop* OR field* OR soil* OR cereal* OR plant* OR food*))

Results: 1,659 articles

*Scopus:*TITLE-ABS-KEY ( ( sustainab* ) AND ( "gen* engi*" OR "gen* manipulat*" OR "gene tech*" OR "gen* modif*" OR "gen* edit*" OR "gen* engineer*" OR "genetically modified organism*" OR "genetically engineered organism*" OR transgenic OR cisgenic OR crispr* OR gmo OR rna* ) AND ( enhanc* OR improv* OR effic* OR innov* OR fortif* OR secur* OR mitigat* OR adapt* OR develop* OR resist* OR control* OR introduc* OR alter* OR creat* OR appl* OR tolera* ) AND ( agri* OR farm* OR crop* OR field* OR soil* OR cereal* OR plant* OR food* ) ) AND PUBYEAR > 2011 AND PUBYEAR < 2023 AND ( LIMIT-TO ( DOCTYPE , "ar" ) ) AND ( LIMIT-TO ( LANGUAGE , "English" ) )

Results: 2,517 articles
